# Evolution of the levels of human leukocyte antigen G (HLA-G) in Beninese infant during the first year of life in a malaria endemic area: using latent class analysis

**DOI:** 10.1186/s12936-016-1131-y

**Published:** 2016-02-09

**Authors:** Tania C. d’Almeida, Ibrahim Sadissou, Gilles Cottrell, Rachida Tahar, Philippe Moreau, Benoit Favier, Kabirou Moutairou, Eduardo A. Donadi, Achille Massougbodji, Nathalie Rouass-Freiss, David Courtin, André Garcia

**Affiliations:** Université Pierre et Marie Curie, Paris, France; UMR216 MERIT “Mère et enfant face aux infections tropicales”, Institut de Recherche pour le Développement, Paris, France; Université Paris Descartes, Paris, France; Centre d’Etude et de Recherche sur le Paludisme Associé à la Grossesse et à l’Enfance (CERPAGE), Cotonou, Benin; Université d’Abomey-Calavi, Cotonou, Benin; UMR Commissariat à l’Energie Atomique et aux Energies Alternatives (CEA), Université Paris Diderot - Paris 7, IMETI Service de Recherches en Hémato-Immunologie, Paris, France; Division of Clinical Immunology, School of Medicine of Ribeirão Preto, University of São Paulo, São Paulo, Brazil

**Keywords:** sHLA-G, Evolution, Groups, Infancy, Malaria, Benin

## Abstract

**Background:**

HLA-G, a non-classical HLA class I antigen, is of crucial interest during pregnancy by inhibiting maternal immune response. Its role during infections is discussed, and it has been described that high levels of soluble HLA-G during childhood increase the risk of malaria. To explore more precisely interactions between soluble HLA-G and malaria, latent class analysis was used to test whether distinct sub-populations of children, each with distinctive soluble HLA-G evolutions may suggest the existence of groups presenting variable malaria susceptibility.

**Method:**

A study was conducted in Benin from 2010 to 2013 and 165 children were followed from birth to 12 months. Evolution of soluble HLA-G was studied by the latent class method.

**Results:**

Three groups of children were identified: one with consistently low levels of soluble HLA-G during follow-up, a second with very high levels and a last intermediate group. In all groups, low birth weight, high number of malaria infections and high exposure to malaria transmission were associated with high level of soluble HLA-G. Placental malaria was not. Presence of soluble HLA-G in cord blood increased the probability of belonging to the highest trajectory.

**Conclusion:**

These results, together with previous ones, confirm the important role of HLA-G in the individual susceptibility to malaria. Assaying soluble HLA-G at birth could be a good indicator of newborns more fragile and at risk of infections during childhood.

## Background

Human Leukocyte Antigen-G (HLA-G) is a non-classical HLA class Ib antigen, with important immune-regulatory functions [1], that differs from classical HLA class I antigens by a low amount of polymorphism and a tissue restricted expression [2, 3]. The physiological HLA-G expression is restricted to fetal tissues such as invasive cytotrophoblast and amnion, and in adults, to immune-privileged organs, cornea, thymus, pancreatic islets, endothelial cell precursors, and erythroblasts. Dendritic cells and macrophages can also express HLA-G [4]. Seven alternative proteins can be generated, four membrane-bound and three secreted isoforms [3–5]. HLA-G is primarily expressed during pregnancy and plays an important role in maternal tolerance of the fetus. Trophoblast cells are protected by HLA-G which inhibit the cytolytic activity of NK cells and protect the semiallogenic fetus from maternal rejections responses [4, 6, 7].

Apart from pregnancy, HLA-G has been shown to be expressed in many types of tumours [8–11]. Tumour cells express HLA-G to escape to immunity and high levels of soluble HLA-G (sHLA-G) have been related with unfavourable outcome of prognosis [12, 13]. In transplantation, HLA-G molecule has been shown to be important for a better allograft acceptance, whereas low serum levels seem to increase the risk of autoimmunity [13, 14] and allergy [15]. If the role of HLA-G with viral infections is admitted [15–18], this association with parasite infections is much less documented [19, 20]. Recently, it was reported that genetic polymorphisms in *HLA*-*G* 3′UTR were associated with susceptibility to human African trypanosomiasis and could influence the clinical and immunological responses directed to *Plasmodium falciparum* [21–23].

A recent work reported that high levels of sHLA-G were significantly associated with a higher incidence ratio of malaria in Beninese children [24] consistent with the fact that the inhibition of immune responses by HLA-G expression could lead to a greater susceptibility to malaria. In this previous analysis, hierarchical mixed models have been used to deal with the repeated measures design. However, mixed models make the assumption that individuals are randomly drawn from a homogeneous population. As a complement it seemed interesting to test whether distinct subpopulations of children are present in the overall population, each with its own parameters, with distinctive sHLA-G evolutions, or trajectories. The identification of trajectories of sHLA-G through the first year of life and risk factors that predispose to, or modify, a particular trajectory may suggest the existence of individual with variable malaria susceptibility. Latent class analysis (LCA) approach is ideally suited to explore this issue [25, 26].

Thus, the main objective of the present work was to explore the existence and characteristics of groups of children with different patterns of soluble HLA-G evolution between birth and 12 months of life in a cohort of Beninese children exposed to malaria, using latent class analysis.

## Methods

### Study design

The work is part of a research programme conducted in southern Benin. The protocol has been widely described elsewhere [24, 27] and will be presented below briefly.

### Site description

The study included nine villages and three health centres: Tori Avamè, Tori Cada and Tori Gare, providing primary healthcare, and holding a maternity for antenatal care and childbirth. The study site was located in a malaria endemic area with an incidence rate of 16.9 % in 2013 [28]. Malaria is transmitted mainly by *Anopheles gambiae s.s.* and *Anopheles funestus* [29]. *P. falciparum* is the most prevalent parasite and infectious peaks occur during the rainy season [28].

### Study population

More than 600 pregnant women were included at delivery from June 2007 to July 2008; they did reside in the villages of the study and give birth in one of the three health centres. Twins, stillbirths and HIV-positive women were excluded. Newborns included were followed-up from birth to 12 months. Among them, 165 mother/infant pairs were selected based on the “placental infection” status of the mother at delivery. Placental infection was defined by the presence of asexual forms of *P. falciparum* in placental blood smears. Population was composed by the totality of mother/infant pairs with placental infection (n = 51) and 114 randomly selected newborns whose mothers were not infected.

### Data collection

Maternal characteristics and data on the course of the current pregnancy were documented through a questionnaire. At delivery, peripheral blood was sampled for soluble HLA-G and haemoglobin measurements. Thick and thin placental smears were performed to determine the existence of a placental *P. falciparum* infection. Cord blood was collected for soluble HLA-G measurements.

At birth, gestational age was estimated using Ballard method [30] and newborn were weighed. Monthly, thick blood smear (TBS) was systematically made (in absence of fever or other clinical sign) to detect asymptomatic malaria infection. Weekly, axillary temperature was measured by trained community health worker. In case of temperature higher than 37.5 °C, the child was lead to the health centre. A questionnaire was filled out and a TBS and a rapid diagnosis test (RDT) were made. Symptomatic malaria cases were defined as fever (>37.5 °C) with RDT and/or TBS positive. TBS obtained monthly or during consultations were stained with Giemsa. Leucocytes and parasites were counted simultaneously until leukocyte or parasite numbers reached 500. A TBS was declared negative if no parasite was found after counting 500 leucocytes. TBSs were read by two laboratory technicians with less than 1 % disagreement. Malaria cases were treated with an artemisinin-based combination therapy (ACT), as recommended in Benin [31, 32]. In case of fever or any clinical sign, mothers were invited to bring their children to health centre where the same protocol was applied. At birth (in cord blood) and at 3, 6, 9 and 12 months old, venous blood was sampled to quantify the level of sHLA-G. Finally, a time-dependent environmental risk of exposure to malaria was assessed for each child by means of a statistical predictive model [33].

### Variable of interest: soluble HLA-G

Assays of sHLA-G have been made by ELISA method. Quantification of sHLA-G correspond to the soluble isoform HLA-G5 and HLA-G1 (after cleavage from the membrane-bound form). Techniques used for these manipulations are extensively described in another study [24]. Soluble HLA-G levels were used as a quantitative variable (after logarithmic transformation) and as a dichotomic one (presence *vs* absence).

### Covariates

From mothers, the following variables were used: Age; parity (primipare vs. multipare); placental malaria; anemia (Hb level before delivery <11 g/dl) and sHLA-G in peripheral blood. From children: low birth weight (LBW) (<2500 g); preterm birth (gestational age <37 weeks of gestation); gender; number of malaria attacks during the follow-up (used as a quantitative variable, or binary variable with two groups: 0 or 1 vs. more than 1); environmental risk of malaria (quantitative time-dependent variable, used as quartiles); sHLA-G level in cord blood and parasite density (PD defined as the number of *P. falciparum* per 100 leucocytes).

### Statistical analysis

Latent class analysis was used to identify the sHLA-G level trajectories and assess their association with the covariates.

A general model’s formulation is:

$$\begin{aligned} y_{it} & = \beta_{0}^{j} + \beta_{1}^{j} Age_{it} + \beta_{2}^{j} Age_{it}^{2} + \beta_{3}^{j} Age_{it}^{3} + \alpha_{1}^{j} X_{1t} \\ &\quad + \cdots + \alpha_{p}^{j} X_{pt} + \varepsilon_{it} \end{aligned}$$

where *y*_*it*_ is the response variable (sHLA-G level of the ith children at age t), the *β*^*j*^ are the coefficients associated to the children’s age in the jth group and the *α*^*j*^ are the coefficients associated to covariates in the jth group (the index t specifies that the covariates can depend on time as the environmental risk of malaria) and ε_it_ the residual variation [ε_it_ ~ Ν (0, σ^2^)].

The posterior probability *π*_*j*_(*z*_*i*_) that a child i with the covariates vector *z*_*i*_ belongs to the group j is: $\pi_{j} (z_{i} ) = \frac{{\exp (z_{i} \theta_{j} )}}{{\sum\nolimits_{j} {\exp (z_{i} \theta_{j} )} }}$ with covariates vector *z*_*i*_ and its corresponding coefficients vector *θ*_*j*_.

The analysis was performed using three steps.

Firstly, each sHLA-G trajectory has been modelled, by means of a polynomial function of time of degree 3, without introducing any covariate. The most appropriate number of trajectories was selected using the Bayesian information criterion (BIC). The group prevalence, that should not be less than 5 % of the total number of the sample, was used as additional criteria, for practical interpretation [34].

Secondly, the effect of the covariates on each trajectory was studied using univariate and multivariate analysis. Only covariates associated with *p* < 0.20 during univariate analysis were entered in the multivariate step. Statistical significance in the final model was set at *p* < 0.05. The effect of covariates on HLA-G trajectories can be identical for each trajectory or different according to them. During this step a particular attention was paid to the stability of the trajectories determined during the first step. Indeed, as mentioned by Nagin [35] the addition of significant covariates to the model should not widely modify the percentage of subjects in each trajectory.

In the final multivariate model each individual was assigned to the trajectory group for which he had the highest posterior probability. Within each group, posterior probability values greater than or equal to 0.7 indicate adequate internal reliability [36]. All these criteria allowed us to make a definitive choice of the appropriate number of classes.

Finally, in the third step, the predictor effect of some covariates to belong to one or more trajectory has been tested. This concerned placental malaria, parity, maternal anaemia, LBW and sHLA-G in cord blood.

All analyses were performed with the Stata^®^ Software, Version 12 (StatCorp LP, College Station, TX, USA), using the syntax dedicated to generalized linear latent and mixed models (GLLAMM).

### Ethics

The study’s protocol was approved by the Ethics Committee of the “Faculté des Sciences de la Santé (FSS)” of Cotonou and the “Institut de Recherche pour le Développement (IRD)” Consultative Ethics Committee. All mothers and fathers were informed during antenatal care visits and meetings conducted in villages before the beginning of the study. Mothers signed informed consent written in French and explained in local language.

## Results

At delivery sHLA-G was measured for all mothers and all cord blood samples (n = 165). During the follow-up, a total of 445 measurements were performed from 3 to 12 months distributed as follows: 109/165 at 3 months, 113 at 6 months, 114 at 9 months and 109 at 12 months. Overall, a total of 775 measurements (mother, cord, 3, 6, 9 and 12 months) were performed. In most cases, an HLA-G measurement was missing because the mother had not attended a quarterly visit. Only 10 children (6.1 %) were present for a single visit.

### Characteristics of the study population

The mean age of the mothers was 26.75 years (SD = 6.37, range 16–49). Most women were multi-gravidae (81.2 %, n = 134), and 40 % were suffering from anaemia at delivery.

The mean birth weight of newborns was 2,964 grammes (SD = 448.31) and 10.9 % (n = 18/165) of children had a LBW. Eighty-eight children (53.33 %) were female and eighteen children were premature. During the follow-up, 50.9 % (n = 84/165) of children had at least one malaria infection, and 10 % more than two. The most infected child had seven infections.

### Soluble HLA-G at delivery

**Fig. 1:**
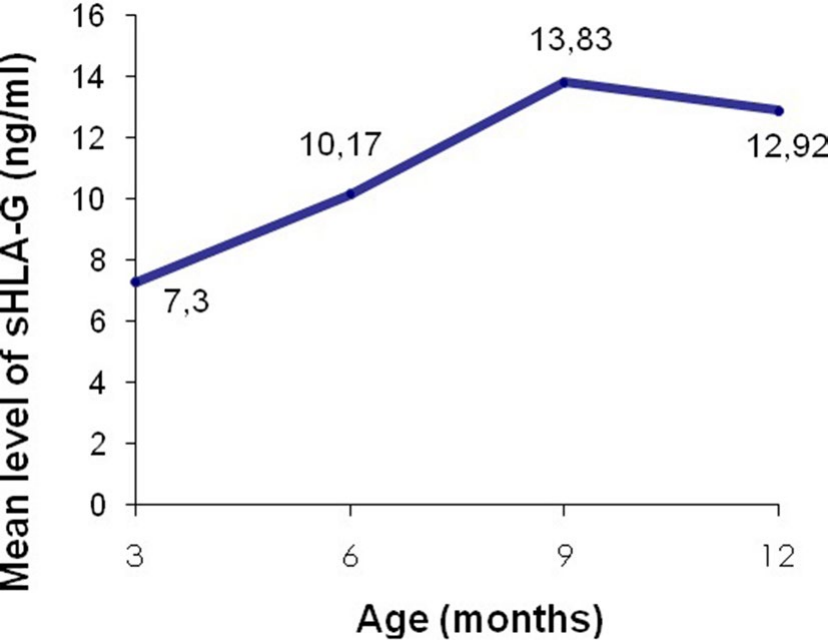
Evolution of the mean level of sHLA-G in newborns in the first year of life

At delivery, 78.2 % of women had detectable levels of sHLA-G (n = 129/165). The mean level in the peripheral blood for all women was 19.12 ng/ml (SD = 25.60, range 0–132.22). Sixty percent of children (n = 99/165) had detectable sHLA-G in cord blood and the mean level was 16.22 ng/ml (SD = 26.83, range 0–128.52). A strong correlation has been described previously between the mother/newborn (cord blood) sHLA-G levels, with a significantly higher level in mother’s blood at delivery [24]. The sHLA-G level increased gradually before stabilizing after 9 months (Fig. 1).

### Trajectory groups of HLA-G

**Fig. 2:**
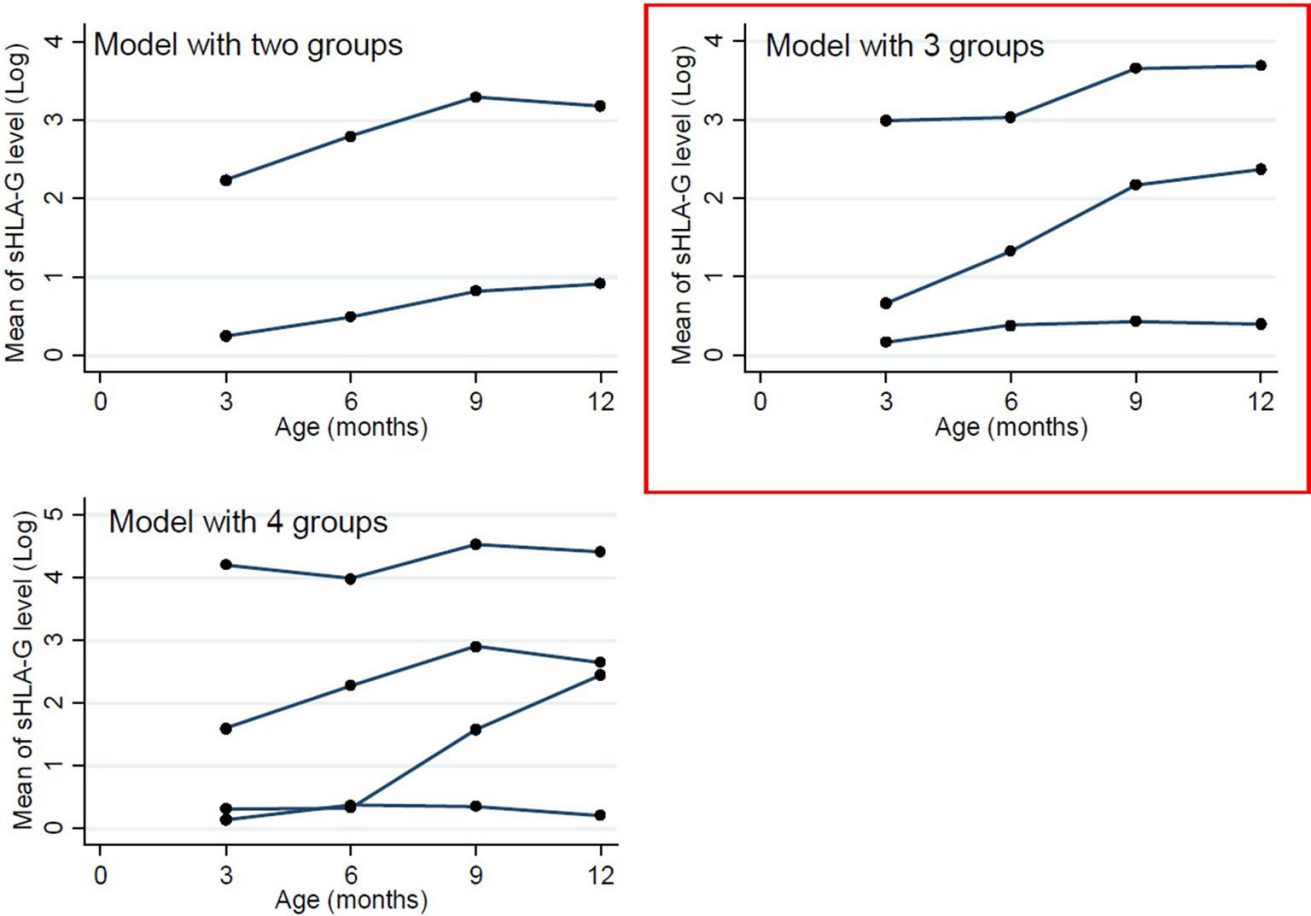
Trajectories of soluble HLA-G for the two-, three- and four-group models

During the first step of analysis, groups of children following the same pattern of sHLA-G have been identified. Using the BIC as criteria, the two-group model showed a higher BIC than both three- and four-group models. The four-group model was selected when compared to the three groups (Table 1). In the three-group model, 16.9 % of the children have high levels of sHLA-G and 53.8 % remained at low levels whereas the third trajectory (29.3 %) was characterized by low levels tending to rise after 6 months. When a fourth trajectory was added, a new intermediate one appeared starting with very low levels until 6 month of age and rapidly rising (Fig. 2; Table 1). From three-group model the following pattern was constantly identified: one trajectory with low levels of sHLA-G (the higher percentage), one with high levels (the lower percentage) and an intermediate one. Adding a new trajectory led to the same trend with the emergence of new intermediate group, representing low percentages of the population. To pursue analysis, the three- and the four-trajectory models only were used (Table 1).

**Table 1:** Distribution of newborns in the two-, three- and four-group models

Trajectories	Model	BIC^a^	Distribution of newborns in groups			
			High (%)	Intermediates… (%)		Low (%)
2	Empty^b^	1494,644	27.2	72.78		
	Full^c^		24.2	75.8		
3	Empty	1464,333	16.9	29.3	53.8	
	Full		21.9	27.2	50.9	
4	Empty	1438,894	7.7	21.9	19.9	50.5
	Full		13.9	15.7	23.1	47.3

^a^A lower BIC indicates a better fitting

^b^With no covariate

^c^With all significant covariates in multivariate analysis

### Effect of covariates on the trajectories

Using the three-group model, placental malaria (p = 0.59), parity (p = 0.31), gender (p = 0.95), prematurity (p = 0.43), maternal anaemia (p = 0.51) and parasite density (p = 0.52) have no significant effect on the dynamics of sHLA-G. In contrast, LBW appeared to be significantly associated with higher levels of sHLA-G (p < 10^−3^). Similarly, detectable sHLA-G level in cord blood, or in mother’s peripheral blood at delivery, was associated with higher levels of sHLA-G from 3 to 12 months of life (p < 10^−3^ in the both case). Having more than one malaria attack during the follow-up was significantly associated with higher levels of sHLA-G (p = 0.008). In the same way, the higher levels of malaria exposure were significantly related to the high levels of sHLA-G (p = 0.01).

**Fig. 3:**
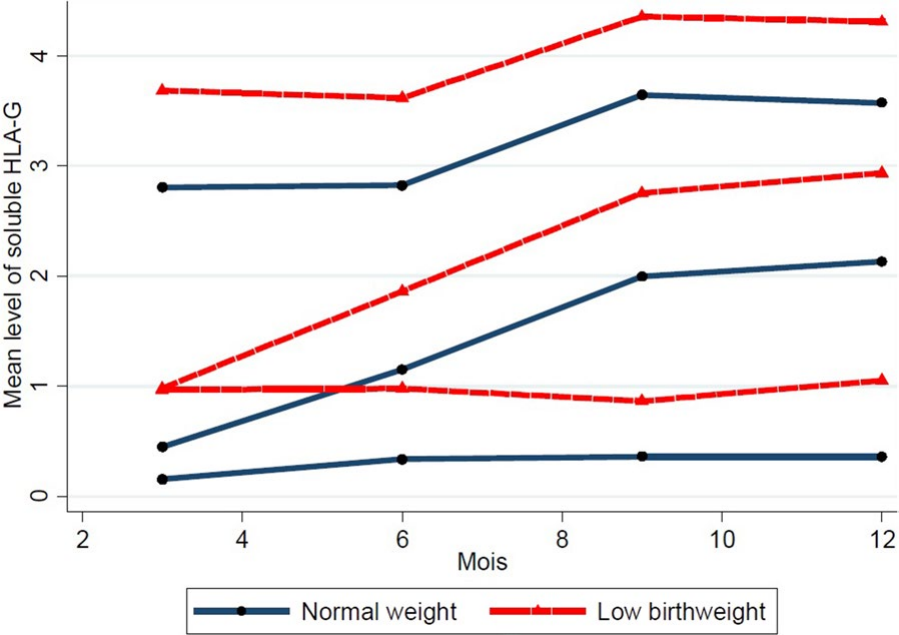
Effect of low birth weight on the evolution of trajectories groups from 3 to 12 months

During multivariate analysis “placental malaria” was forced in the model. The results are presented in Table 2. LBW, presence of sHLA-G in cord blood or in maternal blood at delivery were significantly associated with higher levels of sHLA-G (p < 10^−3^). High exposure to malaria and having more than one malaria infection during the follow-up were significantly associated with high levels of sHLA-G (p = 0.001 and p = 0.002, respectively). In the final model, the proportions of the groups were 50.9, 27.2 and 21.9 %, respectively for the low, intermediate and high trajectories (Table 1). All these covariates have similar effects within each trajectory. As an example, children born with a LBW have significantly higher sHLA-G levels during their first year of live whatever the group they belong (Fig. 3).

**Table 2:** Risk factors of soluble HLA-G evolution from 3 to 12 months in 165 newborns at Tori Bossito, 2007–2010: latent class analysis

Covariates	Univariate estimation^a^	Adjusted estimation^b^	95 % CI	p value
Intercept	–	0.58	0.28–0.88	<10^−3^*
HLA-G in cord blood				
No	–	–		
Yes	0.60	0.42	0.15–0.70	0.002^c^*
Low birthweight (<2500 g)				
No	–	–		
Yes	0.62	0.86	0.42–1.29	*<*10^−3^*
Malaria infections				
≤1	–	–		
>1	0.20	0.33	0.11–0.54	0.003*
Malaria exposure (quartiles)				
Low	–	–		
Middle	0.33	0.43	0.19–0.68	0.001*
High and very high	0.32	0.35	0.13–0.56	0.002*
Placental malaria				
No	–	–		
Yes	–0.07	–0.12	–0.35–0.11	0.36

^a^Univariate estimation represents the coefficient of the univariate regression

^b^Adjusted estimation is obtained after the multivariate analysis

^c^Results were the same, when “HLA-G in cord blood” was replaced by “HLA-G in maternal peripheral blood”. Presence of sHLA-G in maternal blood is highly significantly related to high level of HLA-G in newborns during the first 12 months. We did not introduce together the two covariates in the model because they are highly correlated [24]

Using the four-trajectory model, the same pattern of results was obtained. However, the introduction of LBW resulted in an increase of the proportion of children belonging to the high trajectory from 8 to 17 %, while the intermediate-high trajectory decreased from 22 to 15 %, consistent with a low stability of the four-trajectory model (Table 1).

In the third step of analysis, the role of placental malaria, parity, maternal anaemia, presence of sHLA-G in cord blood and LBW as predictor to belong to either group, were examined. Among them, for the three-group model only the presence of sHLA-G in cord blood was associated with a significantly higher probability to belong to the high or intermediate trajectory (OR = 22.8 and OR = 6.1, respectively). Accordingly, a child born with sHLA-G in cord blood would have a probability of 76.25, 20.40 and 3.35 % to belong to the high, intermediate or the low trajectory, respectively.

When the same analysis was performed using the four-group model, convergence was not achieved, reinforcing the instability of this model. Taken together, the results are consistent with the existence of three-trajectory model. With this model 97 % of children from the low trajectory, 91.7 and 83 % of children from respectively the intermediate and high trajectories were classified as belonging to these groups with a posterior probability greater than 70 %.

Table 3 shows the characteristics of the children and of their mothers in the three groups. LBW children seemed to be more present in the “high trajectory” than in others. Children in this group were born to mothers with detectable sHLA-G at delivery, and the majority had sHLA-G in cord blood.

**Table 3:** Distribution of 165 mother/child pairs in the different groups, 2007–2010

Covariates	High trajectory (n **=** 28/165)	Intermediate trajectory (n **=** 46/165)	Low trajectory (n **=** 91/165)
*Newborns (165)*			
Level of sHLA-G			
3 months	3.29	0.58	0.14
6 months	3.21	1.35	0.31
9 months	3.78	2.32	0.33
12 months	3.88	2.47	0.30
Gender			
Female	17 (60.71 %)	24 (52.17 %)	47 (51.65 %)
Male	11 (39.28 %)	22 (47.83 %)	44 (48.35 %)
Birth weight	2894.52 (SD = 369.30)	3076.63 (SD = 500.10)	2928.57 (SD = 437.05)
Low birth weight	17.86 % (n = 5)	13.04 % (n = 6)	7.69 % (n = 7)
Preterm	10.71 % (n = 3)	6.52 % (n = 3)	13.19 % (n = 12)
Detectable sHLA-G in cord blood	96.43 % (n = 27)	73.91 % (n = 34)	41.76 % (n = 38)
Number of malaria infections during the follow-up	1.11 (SD = 1.31)	0.96 (SD = 1.46)	1.13 (SD = 1.41)
*Mothers (165)*			
Age (years)	26.54 (SD = 6.08)	28.15 (SD = 6.06)	25.78 (SD = 6.48)
Parity	25.00 % (7)	13.04 % (6)	19.78 % (18)
Placental malaria	21.43 % (6)	26.09 % (12)	36.26 % (33)
Detectable sHLA-G in peripheral maternal blood	100.00 % (28)	93.48 % (43)	63.74 % (58)

## Discussion

The present work is complementary to study of Sadissou et al., describing the evolution of sHLA-G in a population of African infants [24]. In the first study, a strong correlation has been shown between mother and cord blood levels of sHLA-G at delivery, as well as an association between LBW and high level of sHLA-G during the first year of life. In addition, high levels of sHLA-G were associated with an increase of the incidence ratio of malaria during the follow-up, but the total number of malaria infections during the follow-up was not associated with the level of sHLA-G.

The present analysis, confirms these results, and provides new understanding by identifying new determinants of sHLA-G evolution. Indeed, although in the first study, the level of sHLA-G increased the risk of infection in the following quarter, any association has not been highlighted between the level of sHLA-G and the overall number of malaria infections during the follow-up. In this complementary analysis, this association is strongly significant. The difference between these two results could be due to the complexity of the effect of malaria infection that cannot be pointed out under the hypothesis that all children belong to the same homogeneous population. Using latent class approach allows hypothesizing that distinct subpopulations of children are presents in the overall population, each with its own characteristics, with distinctive sHLA-G evolutions. This approach permitted us to explore more precisely the complex association between malaria and HLA-G.

The mechanisms by which *HLA*-*G* gene expression is regulated are only partially understood [37]. Concerning infections, HIV or hepatitis, infected patients have higher level of sHLA-G, and it has been suggested that these levels induce tolerance and contribute to immune evasion of virus [38–42]. Although, to date, such hypothesis has not been clarified for malaria; it could be hypothesized that the same phenomenon occurs during malaria infection leading to immune evasion. Indeed, *P. falciparum* could upregulate the HLA-G secretion through the stimulation of cytokines such as IL-10 and IFN-γ. HLA-G is known to inhibit the function of T, NK and B cells through direct interaction with ILT2. Recently, Naji et al. showed that HLA-G inhibits B cell proliferation, differentiation, and immunoglobulin (Ig) secretion in both T cell–dependent and –independent models of B cell activation. HLA-G also acts as a negative B cell regulator in modulating B cell antibodies secretion in a xenograft mouse model [43]. Taken together all these arguments are consistent with the existence of a complex interaction between HLA-G, immunity and *P. falciparum* infection, leading to a lower B cell response. As IgG antibodies play a pivotal role in anti-malarial protection [44–46], the inhibition of IgG specifically directed against *P. falciparum* mediated by HLA-G, not only allows the parasite to escape the immune system but also is responsible of the higher susceptibility to infection showed previously.

However, other factors could influence HLA-G secretion during malaria infection. It has been shown that hypoxia could play a role in HLA-G regulation during pregnancy [47], in some cancer [48] but also in more physiologic but extreme life conditions like altitude [49]. Tissue hypoxia resulting from decreased microcirculatory flow due to parasite sequestration and endothelial dysfunction contributes to the pathogenesis of malaria in children [50, 51]. The results could be consistent with the fact that malaria related hypoxia is already present very early in life and associated with an increase of sHLA-G levels, and consequently strengthening the inhibition of immunity.

More than 60 % of the children born with a LBW belong to the high and intermediate trajectories. It is widely accepted that birth weight is a key indicator of fetal and neonatal health [52–54] and overall 60–80 % of infants who die during the neonatal period are children born with LBW [55]. The association between LBW and HLA-G has already been shown in this same cohort using mixed model [24]. However, the present approach allows pointing out a very interesting aspect, which has not been demonstrated by previous analysis. Indeed, children born with detectable HLA-G in their cord blood have a greater probability to belong to the higher or intermediate trajectories than other children, and hence be concerned by LBW. Considering the frailty of LBW children, the detection of such high-risk individuals could have huge public health consequences. Taking into account the strong correlation between sHLA-G in cord blood and in mother’s blood at delivery, the mother’s sHLA-G level could be considered as a potential marker of high-risk newborn. Soluble HLA-G has already been proposed as predictor of in vitro fecundation outcome [56], but also as biomarker of allergy [57], allograft and cancers [58, 59]. A follow-up of pregnant women and of their offspring over 2 years is now ongoing in Benin to test whether the sHLA-G level early in pregnancy is also correlated with HLA-G level in cord blood or with infectious morbidity during the first 2 years of life.

Latent class analysis approach is more and more frequently used in biomedical research [60]. It is an extension of mixed models that assume the presence of several groups of subjects (latent groups) sharing a particular evolution trajectory of some attribute. This approach allows a better understanding of the phenomenon under study and the formulation of new questions or hypotheses [61, 62]. This methodology is part of mixture modelling, a wide data analysis approach allowing studying unobserved heterogeneity in a population [60]. The question of the number of trajectories remains of great importance and to date, there is no single statistical test to determine the correct number of latent classes [63]. The selection of the best model needs to take into account statistical measures of model fitting but also the interpretation of the output, in particular the relevance of the different groups, based on the literature or the expert’s knowledge. BIC was used as statistical criteria although others can be proposed. Bootstrap Likelihood Ratio test (BLRT) has been proposed as being more powerful. However, BLRT needs an increased computation time and finally the authors recommended using BIC as the first step [64]. In the data, using BIC would lead to choose a model with four trajectories instead of three. Both model have the same interpretation since the two intermediate trajectories in the four-group model share the same evolution, i.e. high level trajectory, low level trajectory and intermediate group(s) starting with low levels of sHLA-G and then growing up. However, models with more than three trajectories seem to present a lower stability. Finally, to deal with the possibility and complexity of these population mixture models, it could be of interest to pursue this exploration by using different methods in different populations.

A genetic association between *HLA*-*G* and malaria has already been pointed out. Indeed, it has been shown that some polymorphisms of the *HLA*-*G* 3’UTR were associated with variable risk of infection [22], but also with antibody responses against *P. falciparum*. These associations have been identified in another population from Senegal. It has also been demonstrated that a strong molecular signature of balancing selection at *HLA*-*G* 5’URR exist and that this region may certainly be the direct target of selection [65]. These results strengthen the interest of this gene and of sHLA-G as potentially involved in differential susceptibility to malaria.

## Conclusion

Summary, this study allowed a better understanding of the complex interaction between HLA-G and malaria infection and pointed out the potential interest of sHLA-G as marker of frailty.

## Abbreviations

HLA-G: human leukocyte antigen-G
sHLA-G: soluble human leukocyte antigen-G
LCA: latent class analysis
TBS: thick blood smear
RDT: rapid diagnosis test
ACT: artemisinin-based combination therapy
LBW: low birth weight
PD: parasite density
BIC: Bayesian information criterion
GLLAMM: generalized linear latent and mixed models
SD: standard deviation
OR: odd ratio
NK: natural killer
